# Ten tips to control blood pressure in haemodialysis patients

**DOI:** 10.1093/ckj/sfag128

**Published:** 2026-04-28

**Authors:** Shanmugakumar Chinnappa, Amaryllis H Van Craenenbroeck, Evangelia Dounousi, Beatriz Fernandez-Fernandez, Fotini Iatridi, Nejc Piko, Johannes Stegbauer, Claudia Torino, Liffert Vogt, Jose Manuel Valdivielso, Patrick B Mark

**Affiliations:** Department of Nephrology, Doncaster and Bassetlaw Teaching Hospitals NHS Trust, Doncaster, UK; Leeds Institute of Cardiovascular and Metabolic Medicine (LICAMM), University of Leeds, Leeds, UK; Department of Nephrology, University Hospitals Leuven, Leuven, Belgium; Department of Microbiology, Immunology and Transplantation, Nephrology and Renal Transplantation Research Group, KU Leuven, Leuven, Belgium; Department of Nephrology, University Hospital of Ioannina, Ioannina, Greece; IIS-Fundacion Jimenez Diaz UAM, Madrid, Spain; Department of Medicine, School of Medicine, Universidad Autónoma de Madrid, Madrid, Spain; RICORS2040, Madrid, Spain; First Department of Nephrology, Hippokration Hospital, Aristotle University of Thessaloniki, Thessaloniki, Greece; Department of Dialysis, Clinic for Internal Medicine, University Medical Centre Maribor, Maribor, Slovenia; Department of Nephrology, Medical Faculty, University Hospital Düsseldorf, Heinrich-Heine-University Düsseldorf, Düsseldorf, Germany; Cardiovascular Research Institute Düsseldorf, Medical Faculty, Heinrich Heine University, Germany; Clinical Epidemiology of Renal Diseases and Hypertension Unit, Institute of Clinical Physiology, Reggio Calabria, Italy; Department of Internal Medicine, Section of Nephrology, Amsterdam, The Netherlands; Amsterdam Cardiovascular Sciences, Amsterdam, The Netherlands; Vascular and Renal Translational Research Group, UDETMA, RICORS2040 del ISCIII, IRBLleida, University of Lleida, Lleida, Spain; RICORS2040, Madrid, Spain; School of Cardiovascular and Metabolic Health, University of Glasgow, Glasgow, UK; Renal and Transplant Unit, Queen Elizabeth University Hospital, Glasgow, UK

**Keywords:** cardiovascular, chronic haemodialysis, dry weight, hypertension, physical activity

## Abstract

Hypertension is extremely common among individuals with kidney disease undergoing haemodialysis, with an estimated prevalence exceeding 80%. However, uncertainties remain on how best to manage hypertension in this group. This review discusses in detail the diagnosis, mechanism, and more importantly the management of hypertension in patients on haemodialysis presented as ‘10 tips’ for easy application in day-to-day clinical practise.

## INTRODUCTION

**Figure 1:**
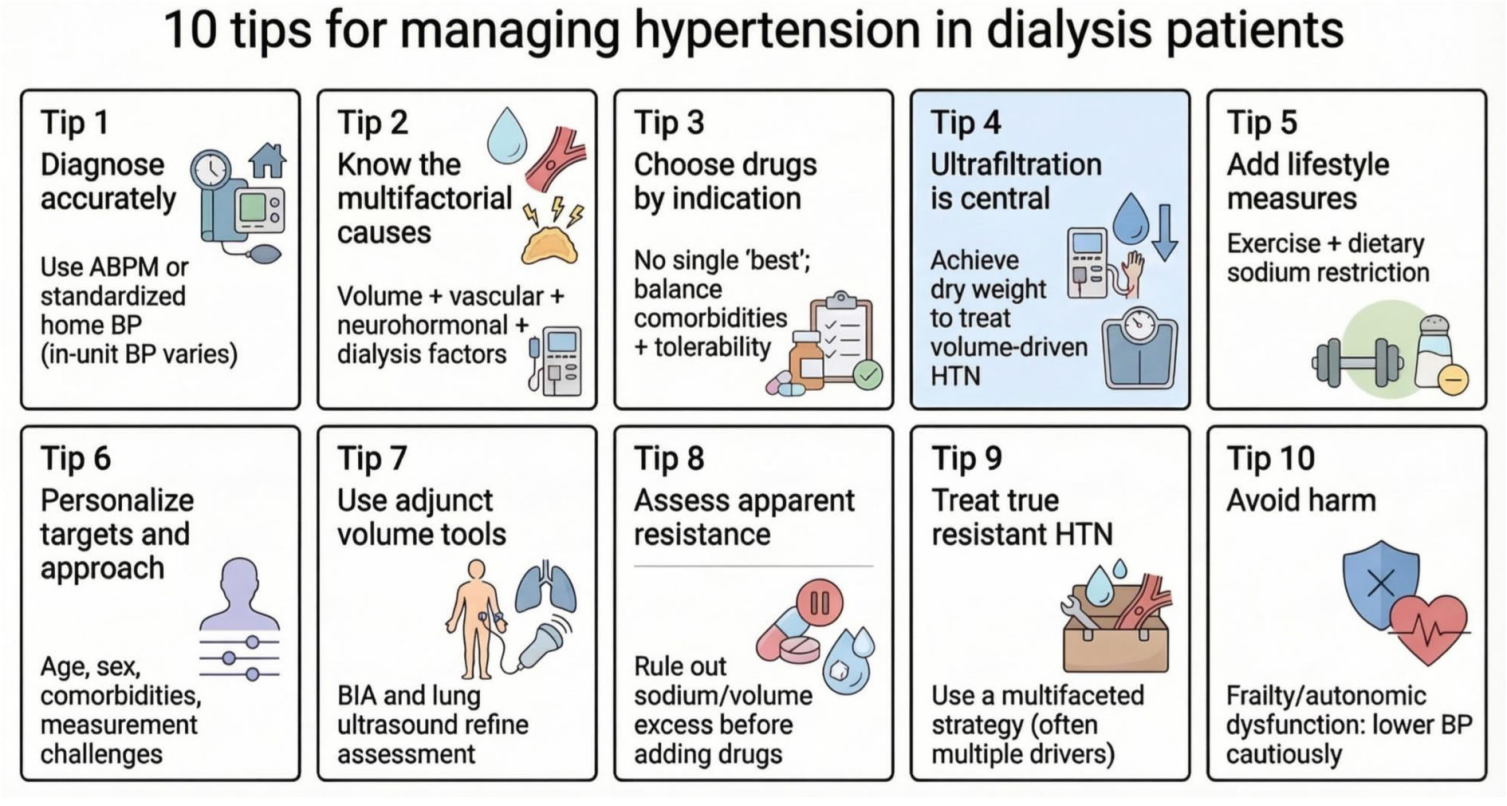
Overview: 10 tips for managing hypertension in dialysis patients.

In chronic kidney disease (CKD), hypertension can be the cause and the consequence, and also a compounding factor in causing cardiovascular (CV) disease complications. Blood pressure (BP) homeostasis is highly complex in haemodialysis (HD) patients and the management strategies in general population or early CKD may not be readily transferable to this cohort. As there are only limited number of clinical trials to guide therapy in this setting, good BP management in HD depends on a clear understanding of the underlying mechanisms, the complexities in diagnosis and setting treatment targets, and judicious application of pharmacological and nonpharmacological treatment strategies. This review aims to equip the clinician with such knowledge conveniently packaged in the form of 10 tips to help deliver personalized care in managing hypertension in HD patients (Fig. 1).

## TIP 1: HOW TO DIAGNOSE HYPERTENSION IN HD PATIENTS?

In HD patients, the diagnosis of hypertension is routinely made when predialysis or postdialysis BP is >140/90 mmHg and >130/80 mmHg, respectively [1]. However, these peridialytic measures have a high misclassification rate [2–4] and are no longer recommended by the last guidelines promoted by European Renal Association (ERA) and European Society of Hypertension (ESH) [5].

In the general population, BP varies mainly with circadian rhythms [6]. In HD patients, BP fluctuates with fluid shifts during and between sessions [2] and is further shaped by a heterogeneous range of factors (*vide infra* Tip 2), so single measurements only reflect a moment within this dynamic cycle.

Ambulatory Blood Pressure Monitoring (ABPM) remains the gold standard for diagnosing hypertension in HD patients [2], and the only method for identifying the nondipping nocturnal BP pattern [7]. An average value of ABP ≥130/80 mmHg, measured for 44 h (starting after the dialysis session) is suggestive of hypertension [5]. However, the application of ABPM is still limited [8], with discomfort frequently reported during the measurements hindering its application in these patients [9].

When ABPM cannot be applied, home BP represents a valid alternative [3]. Due to the BP fluctuations observed in HD patients, standardized measurements [10] should be performed twice a day, over six nondialysis days [5]. Hypertension is diagnosed when the average value of home BP is ≥135/85 mmHg. If home BP or ABPM is not feasible, the diagnosis of hypertension can be made when the average of three standardized office BP measurements, taken during a mid-week nondialysis day, is ≥140/90 mmHg [5, 10].

Further research is needed to provide optimal protocols for BP measurements in HD patients. Due to the current scarcity of evidence, the targets still used for diagnosis and management of hypertension in HD patients come from very old guidelines [1, 11, 12], with the newer ones preferring not to give suggestions [13] or being less prescriptive [14] (Table 1).

**Table 1:** Definition of hypertension in dialysis patients as outlined in clinical practise guidelines [14, 15].

Guidelines	Year	Definition
KDOQI 2005	2005	Predialysis BP of >140/90 mmHg
		Postdialysis BP of >130/80 mmHg
KDOQI 2015 update	2015	No target defined, citing paucity of clinical trial data
CSN 2006	2006	Predialysis BP of >140/90 mmHg
ERA-EDTA/ESH 2017	2017	No recommendation can be made on the basis of peridialytic BPHome BP: an average BP of ≥135/85 mmHg for measurements collected in the morning and evening over six nondialysis days (covering a period of 2 weeks)ABP: an average BP of ≥130/80 mmHg over 24-h monitoring during a mid-week day free of HD. Whenever feasible, ABP monitoring should be extended to 44 h (covering a whole mid-week dialysis interval)Office BP of ≥140/90 mmHg taken in a mid-week day free of HD (when neither ABP nor home BP measurements are available)
KSN 2021	2021	Inconclusive, insufficient evidence to assign optimal BP target for HD patients
UKKA	2025	Recommends ABPM as the gold standard technique; however, concedes that there is insufficient evidence to assign BP cutoff to diagnose hypertension.

CSN, Canadian Society of Nephrology; ERA–EDTA, European Renal Association–European Dialysis and Transplantation Association; KDOQI, Kidney Disease Outcomes Quality Initiative; KSN, Korean Society of Nephrology; UKKA, UK Kidney Association.

## TIP 2: BE MINDFUL OF THE COMPLEX PATHOGENESIS OF HYPERTENSION IN HD PATIENTS

Understanding the aetiology of hypertension in chronic HD patients is key to explaining their high CV risk. Hypertension arises from a multifactorial interplay of extracellular volume expansion, vascular and endothelial dysfunction, neurohormonal activation, and dialysis-related haemodynamic stress.

Volume overload is a central determinant. In kidney failure, impaired sodium and water excretion lead to chronic extracellular fluid expansion, increasing venous return, cardiac preload, and stroke volume, thereby elevating BP [16, 17]. The repetitive cycle of fluid removal and re-accumulation between sessions prevents true euvolaemia and sustains hypertension. Persistent overload promotes left ventricular hypertrophy/stiffness and baroreflex impairment [18, 19], which further perpetuate hypertensive states. Detecting subclinical overload remains challenging, prompting use of tools like bioimpedance and lung ultrasound (LUS; *vide infra* Tip 7) [20].

Vascular dysfunction is nearly universal in HD patients [21]. Medial calcification, smooth muscle hypertrophy, and matrix remodelling cause arterial stiffening, increasing pulse-wave velocity and systolic pressure, while hypertension accelerates stiffness in a vicious cycle [22]. Endothelial dysfunction, driven by reduced nitric oxide, oxidative stress, uremic toxins, mineral metabolism alterations, and inflammation, favours vasoconstriction [23].

Neurohormonal dysregulation adds to this burden. Chronic activation of the renin–angiotensin–aldosterone system (RAAS) and sympathetic nervous system (SNS) is consistently observed [24, 25]. RAAS promotes vasoconstriction, sodium retention, and vascular remodelling, while SNS overactivity, linked to renal afferent signalling and chemoreceptor stimulation, increases systemic resistance and arrhythmogenic risk. Importantly, SNS hyperactivity persists independently of residual kidney function, amplifying CV strain [26]. While direct pharmacological targeting of RAAS and SNS is discussed in Tip 3, awareness of their chronic activation helps explain why BP may remain elevated even after optimal volume control and guides a more realistic expectation of treatment outcomes.

Dialysis-related factors further modulate BP. Rapid ultrafiltration and osmotic shifts can trigger intradialytic hypertension (IDHTN) via endothelial dysfunction and endothelin-1-mediated vasoconstriction, particularly in patients with extracellular fluid overload [27]. Repeated exposure to these perturbations and large interdialytic fluid gains may reduce baroreceptor sensitivity and sustain interdialytic hypertension [28]. Dialysate sodium and temperature influence vascular tone and autonomic activity, shaping intra- and interdialytic BP variability [29].

In summary, hypertension in HD patients is multifactorial, and understanding the underlying pathophysiology supports a nuanced, individualized approach to BP management.

## TIP 3: CONSIDER PERSONALIZING ANTIHYPERTENSIVE THERAPY BASED ON INDICATION AND TOLERABILITY

Perhaps surprisingly, there are limited outcome trials to inform selection of antihypertensive agents in patients treated with HD. The totality of the evidence was comprehensively summarized in the previous ERA/ESH consensus document on the topic [5]. Meta-analyses of the published trials on treating dialysis patients with antihypertensive agents suggest that treatment is associated with reduced CV mortality and morbidity compared to placebo or no treatment [30]. It should be noted that trials of these antihypertensive agents were often trials of the agents for a number of indications (e.g. cardiomyopathy, hypertension, CV protection, etc.) rather than specifically testing that agent for BP control.

Perhaps the most convincing data exist for β-blockers, mainly evidenced by the Hypertension in hemodialysis patients treated with atenolol or lisinopril trial (HDPAL Trial) in 200 hypertensive HD patients, which demonstrated that compared to the angiotensin converting enzyme inhibitor (ACEi) lisinopril, the β-blocker atenolol (both administered postdialysis) led to numerically lower BP, requiring fewer additional antihypertensive agents and less need for ultrafiltration [31]. Importantly, the trial was terminated early due to the statistically significant superiority of atenolol over lisinopril for prevention of serious CV events [myocardial infarction (MI), stroke, hospitalized heart failure, and CV death]. Although often mooted, a large multicentre outcome trial of β-blockers in dialysis has proved challenging to undertake. It is important to consider the differences in renal clearance and dialysability between different β-blockers. A large retrospective cohort study suggests that highly dialysable β-blockers are less cardioprotective, possibly due to a lack of intradialytic protection against arrhythmia and therefore nondialysable agents are preferable [32].

Although there are multiple theoretical benefits of using renin–angiotensin system inhibition (RASi) in dialysis patients mainly for cardioprotection, there are limited clinical trial data to support this with one example being the Fosinopril in Dialysis trial, in which 397 HD patients were randomized to receive fosinopril or placebo for a follow-up period of 48 months [33]. Although therapy with fosinopril resulted in a significant reduction in predialysis BP versus placebo, there were no differences in the CV outcome between groups. The recent large CV outcome randomized controlled trials (RCTs) of mineralocorticoid antagonism (MRA) in dialysis patients (Aldosterone Blockade for Health Improvement Evaluation in End-Stage Renal Disease and Aldosterone Antagonist Chronic HEModialysis Interventional Survival Trial) did not demonstrate benefit with spironolactone compared to placebo on CV outcomes, with significantly greater risk of hyperkalaemia with spironolactone [34]. These definitive results contrast with meta-analyses of smaller underpowered trials of MRA in this patient group [34].

Calcium channel blockers are potentially attractive agents, based on their lack of effect on potassium, unlike β-blockers and RASi. However, their propensity to cause oedema limits their use. Like other agents, there is paucity of data to inform their impact on outcomes. However, one RCT in 251 hypertensive HD patients demonstrated potential benefit of amlodipine compared to placebo on CV outcomes, although the overall number of events was small [35].

In summary, given the overall lack of demonstrative outcome data clearly favouring one specific agent in appropriately powered CV outcome trials in dialysis patients, the choice of antihypertensive agent should take into account the tolerability and potential indications for that agent while considering other comorbidities such as heart failure, rather than solely focusing on the effect of any specific drug class on BP targets.

## TIP 4: RECOGNIZE THE CENTRAL ROLE OF ULTRAFILTRATION

One cannot overstate the role of attaining euvolaemia to achieve normotension in HD patients as volume overload is the primary cause of hypertension in this patient group (*vide supra* Tip 2). In practical terms euvolaemia is equated to dry weight, which Agarwal *et al*. defined as ‘the lowest tolerated postdialysis weight achieved via gradual change in postdialysis weight at which there are minimal signs or symptoms of hypovolaemia or hypervolaemia’ [17]. Dry weight can be estimated using physical examination findings such as peripheral oedema. However, this can be imprecise [36]. Adjunctive tools such as bioimpedance analysis (BIA) and LUS may help define dry weight (*vide infra* Tip 7).

After estimation, achieving dry weight should proceed incrementally over weeks. Although a safe rate of ultrafiltration is difficult to define and generalize, we recommend a rate <10 ml/min/kg as observational studies have shown that a rate >10 ml/min/kg has been associated with complications including increased risk of all cause and CV mortality [37, 38].

In an RCT involving 150 HD patients, a gradual reduction of dry weight by 1 kg over 8 weeks resulted in an additional decrease in SBP of 7 mmHg (on ABPM) [16]. If conventional short dialysis session is a limiting factor, one should consider longer or more frequent sessions [39].

Observational data from >700 patients had demonstrated that dry weight attainment over 2 months resulted in commensurate drop in BP, and a further slow and progressive reduction in BP over a 12-month period even without further reduction in extracellular fluid. This additional benefit was attributed to delayed regression in peripheral vascular resistance that developed in response to chronic fluid overload [40]. In addition, dry weight attainment can also lead to beneficial cardiac effects such as reduction in left ventricular mass, chamber dimension, and filling pressures [41]. Conversely, failure to achieve dry weight has been shown to be strong predictor of impending hospital admission [42].

It is important to avoid overzealous ultrafiltration, as efforts to correct resulting hypotension with intradialytic saline infusion or sodium profiling can be counterproductive. Such interventions may lead to a positive sodium balance and exacerbate hypervolaemia [43].

Finally, achieving dry weight should involve a combination of ultrafiltration and appropriate lifestyle interventions, such as salt and fluid restriction (*vide infra* Tip 5). These strategies may even result in successful withdrawal of antihypertensive drugs as demonstrated by observational data from centres practising strict volume control [40].

## TIP 5: INCORPORATE LIFESTYLE INTERVENTIONS

Lifestyle interventions have a crucial role in both preventing and controlling hypertension and they should complement evidence-based pharmacological and volume-based therapies, particularly in patients with poorly controlled hypertension. All guidelines on BP management (ESH, International Society of Hypertension, and European Society of Cardiology) agree that they should be part of first-line antihypertensive treatment, independent of the grade of hypertension and associated CV risk [44, 45].

All patients with CKD, including HD, should reduce sedentary time and engage in at least light activity throughout the day. Moreover, they are advised to strive for at least 150 min a week of moderate intensity, or to a level compatible with their CV and physical tolerance [46]. For dialysis patients, most exercise regimens have proven benefits across multiple domains of wellbeing, including quality of life, physical fitness, and CV risk factors such as hypertension. Most convincing evidence for the BP lowering effect is on combined aerobic and resistance intradialytic exercise, which has been shown to be the most effective intervention for improving cardiorespiratory fitness and reducing (diastolic) BP in comparison with usual care [47]. Moreover, a meta-analysis of intradialytic exercise programs found clinically meaningful improvements of several physiological measures of CV health such as pulse wave velocity, a measure of arterial stiffness [48].

**Figure 2:**
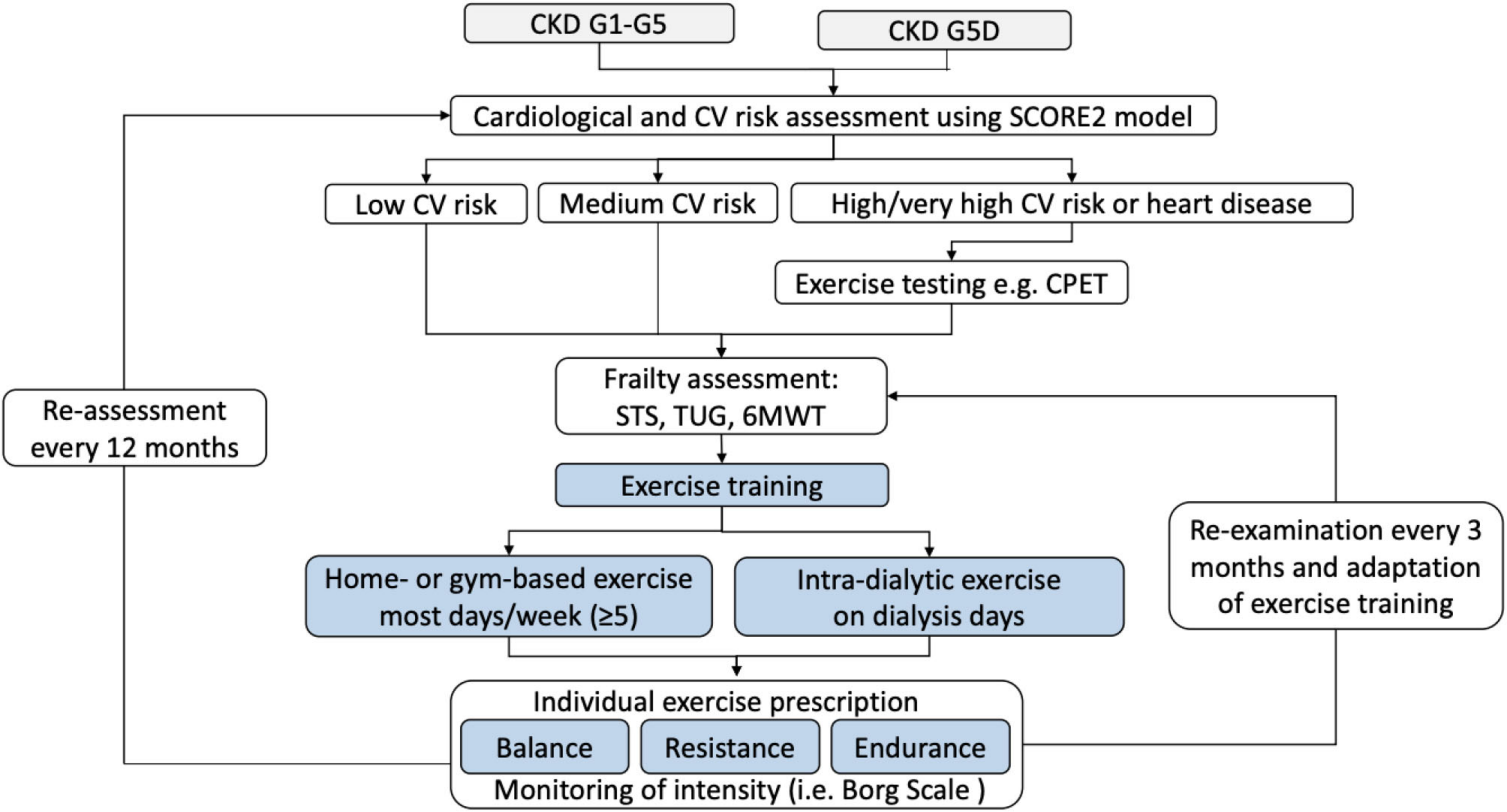
Algorithm for exercise prescription in patients with chronic kidney disease and kidney failure treated by dialysis. CKD, chronic kidney disease; CV, cardiovascular risk; CPET, cardiopulmonary exercise testing; STS, sit-to-stand test; TUG, timed up and go test; 6MWT, 6-minute walk test.

Overall, there is limited evidence on how best to prescribe exercise treatment and ensure safety in HD patients, with concomitant risk factors and comorbidities. With intradialytic programs, exercise could be envisioned as a regular part of the patient’s treatment schedule, with supervised activities ensuring safety and adherence. On the other hand, home-based exercise offers flexibility and convenience, and adherence could be improved with the use of digital health interventions for promoting healthy lifestyle [49] also in HD patients [50]. Moreover, CV risk, frailty, and multimorbidity should be taken into account and, ideally, a multidisciplinary team should be involved in personalized exercise prescription [51] (see Fig. 2).

Dietary sodium restriction reduces thirst, helps fluid restriction, reduces interdialytic weight gain, and thereby facilitates BP control [52]. According to the KDIGO Blood Pressure Work Group, the Dietary Approaches to Stop Hypertension-type diet or use of potassium-rich salt substitutes may not be appropriate for patients with CKD [53]. This caution might be particularly relevant for individuals undergoing HD, with the KDOQI 2020 Clinical Practice Guideline recommending adjusting dietary potassium intake to maintain serum potassium within the normal range [54]. Nevertheless, further research is warranted to clarify efficacy, safety, and feasibility of potassium-rich diets in this population [55].

Other lifestyle interventions for BP control also apply for the dialysis population: optimize weight management, stop tobacco smoking, restrict free sugar consumption, and limit alcohol intake [45].

## TIP 6: REMEMBER TO PERSONALIZE CARE: ONE SIZE DOES NOT FIT ALL

Individualized management of hypertension in HD patients is vital for optimal outcomes. A standardized approach may be inadequate due to patient-specific factors such as age, sex, clinical characteristics, and comorbidities. Notably, nearly 50% of the European dialysis population is aged 65 years or older [56], necessitating consideration of accelerated vascular ageing, increased arterial stiffness, endothelial dysfunction, and altered neurohormonal regulation when treating elderly patients [5].

Recent studies indicate that both sexes exhibit similar hypertension prevalence among dialysis patients; however, men tend to have higher ABP and central BP levels and poorer control rates compared to women [57, 58]. Current practise focuses on optimizing volume status, dietary sodium restriction, individualized dialysate sodium concentration, and appropriate use of antihypertensive medications, regardless of sex [3].

Nonpharmacological management, such as achieving dry weight and adjusting dialysate sodium concentration, should be tailored to individual patient needs. Dry weight attainment is discussed above (Tip 4). Customizing dialysate sodium concentration in dialysis patients involves individualizing the prescription based on the patient’s predialysis plasma sodium and clinical factors such as volume status, interdialytic weight gain, BP, and haemodynamic stability, rather than using a standard concentration for all patients. This approach aims to minimize the sodium gradient between plasma and dialysate, thereby reducing diffusive sodium loading or depletion [59, 60].

Pharmacological treatment also demands a personalized approach (discussed in detail in Tip 3). Common antihypertensive agents for this population—such as β-blockers, calcium channel blockers, and ACEis/ARBs should be selected with attention to residual renal function, comorbid conditions (e.g. diabetes, CV disease, and heart failure), frailty, intradialytic symptoms, and laboratory findings (e.g. hyperkalaemia).

Measuring BP in HD patients with multiple AV fistulas is challenging. Avoid using any arm with a functioning AVF; arms with old, ligated, or thrombosed AVFs are generally safe. If both arms are unusable, measure BP in the lower extremities with a properly sized cuff. For difficult cases, validated noninvasive devices like wrist monitors (at heart level) may be considered.

The absence of comprehensive guidelines and widely accepted BP targets in this patient group emphasizes the need for personalized care and reinforces the principle that ‘one size does not fit all’ in the management of hypertension in HD patients.

## TIP 7: MAKE USE OF ADJUNCTIVE ASSESSMENT TOOLS

**Figure 3:**
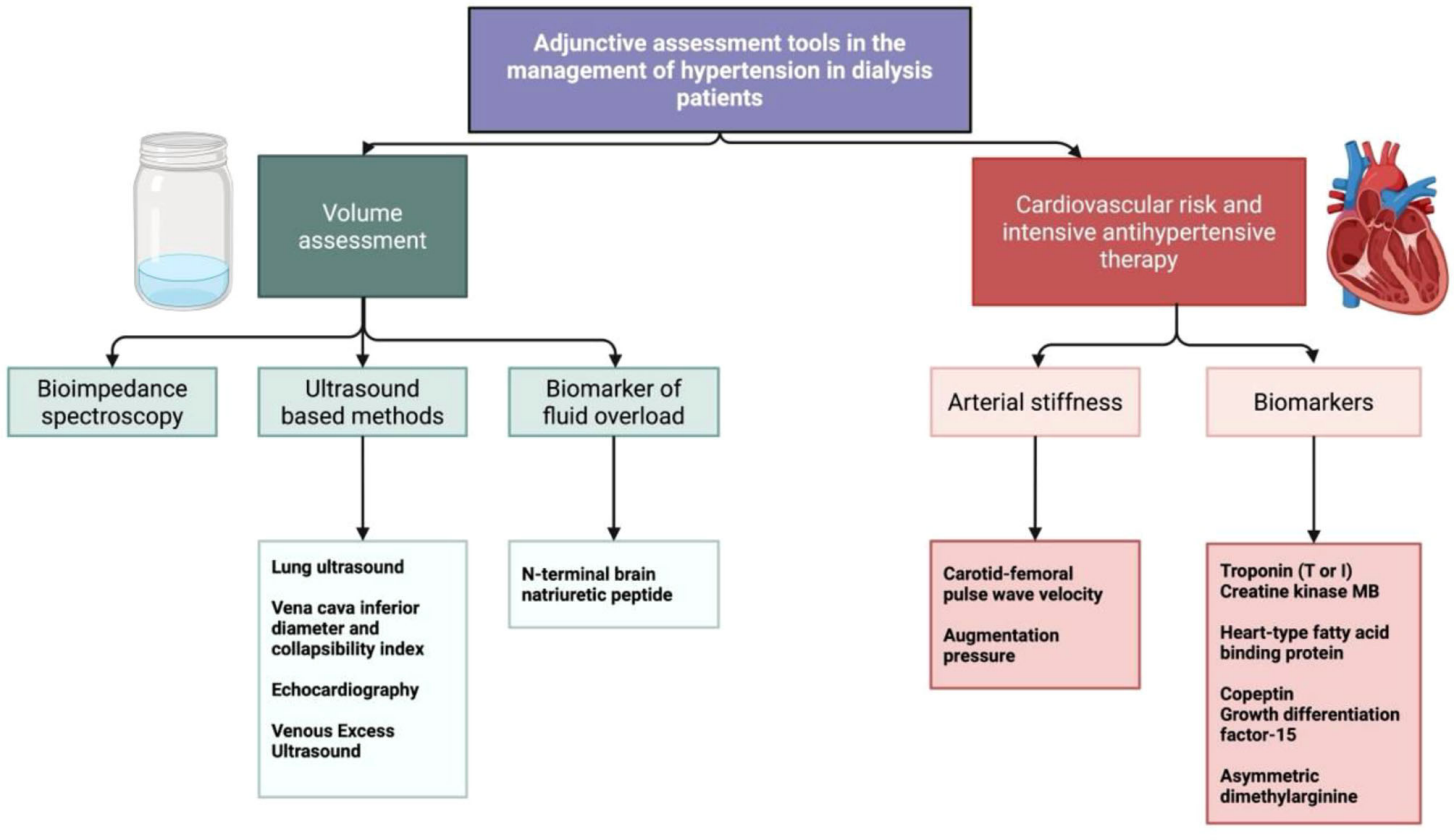
Adjunctive assessment tools in the management of hypertension in dialysis patients.

Several adjunctive strategies show promise for improving hypertension control in this population (Fig. 3). BIA noninvasively measures tissue resistance to electrical currents and can determine volume status [61]. In addition to identifying extracellular fluid overload, BIA can help distinguish fluid-related hypertension from other mechanisms and guide progressive dry weight adjustment. In a randomized trial of 156 HD patients, bioimpedance-guided fluid assessment improved fluid control and BP control, and reversed left ventricular hypertrophy (LVH) and arterial stiffness [62]. In a 2.5-year randomized trial of 131 HD patients, bioimpedance-guided dry weight management led to greater reductions in BP, arterial stiffness, fluid overload, and all-cause mortality [63].

Emerging adjunctive techniques are increasingly recognized for their potential to enhance volume and hypertension assessment in HD patients, complementing standard clinical evaluation.

- **LUS:** Detects pulmonary congestion via B-lines and consistently outperforms standard assessment in guiding dry-weight reduction and improving BP control [64–66].
- **IVC ultrasound:** Assesses central venous pressure and intravascular volume shifts; accuracy improves when combined with other modalities [67, 68].
- **Doppler echocardiography:** Evaluates cardiac filling pressures, with tools such as VEXUS refining the assessment of systemic venous congestion [69, 70].
- **Arterial stiffness measures:** Indices such as carotid–femoral pulse wave velocity provide insight into vascular contributors to hypertension [71, 72].
- **NT-proBNP**: Serves as a biomarker of extracellular fluid burden and strengthens multimodal approaches to volume assessment [73].

Accurate extracellular volume assessment is essential for hypertension management in dialysis, but no single gold standard exists; adjunctive tools should therefore complement clinical evaluation to better guide volume assessment and hypertension phenotyping [74].

## TIP 8. HOW TO EVALUATE AND MANAGE APPARENT TREATMENT RESISTANCE

Resistant hypertension is defined by BP above target despite use of ≥3 classes of antihypertensive agents at maximally tolerated doses (one being a diuretic in nonanuric) or requirement of ≥4 agents to reach control [75].

The definition of ‘true resistant hypertension (tRH)’ is persistent elevation of BP after excluding apparent resistance (arHTN) by confirming patient adherence to medication, ensuring accurate measurement techniques, and implementing an optimal treatment regimen. However, this assessment presents challenges in patients undergoing HD or peritoneal dialysis (PD), as target BP remains controversial and highly variable across studies [76]. Additionally, a large meta-analysis involving 71 353 HD patients reported a medication nonadherence prevalence of 37% (95% CI, 27%–47%) [77].

Definition of tRH is complicated in dialysis population, as it can be mediated by altered volume status and timing of BP measurement (pre- vs postdialysis BP vs ambulatory/interdialytic) so, there is an unmet need for properly differentiating patients with arHTN (to avoid polypharmacy or delay treatable causes such as volume overload, dry weight, and sodium balance) from those with tRH who will need more intensive treatment [78].

Volume overload and positive sodium balance are the main drivers of arHTN in dialysis population (discussed above). Other conditions related to arHTN can be arterial stiffness (difficult to modify with antihypertensive therapy) [79] or erythropoietin-induced hypertension [80].

In addition, we must also differentiate tRH from IDHTN, defined as paradoxical rise of BP during or immediately after HD, usually systolic BP increase >10 mmHg from pre to postdialysis in a minimum of four–six consecutive sessions [81]. IDHTN is possibly mediated by extracellular volume overload, disbalances in ultrafiltration rate, sodium and calcium in dialysate, sympathetic activation, endothelial dysfunction, or RAAS activation with implications on mortality [82].

**Figure 4:**
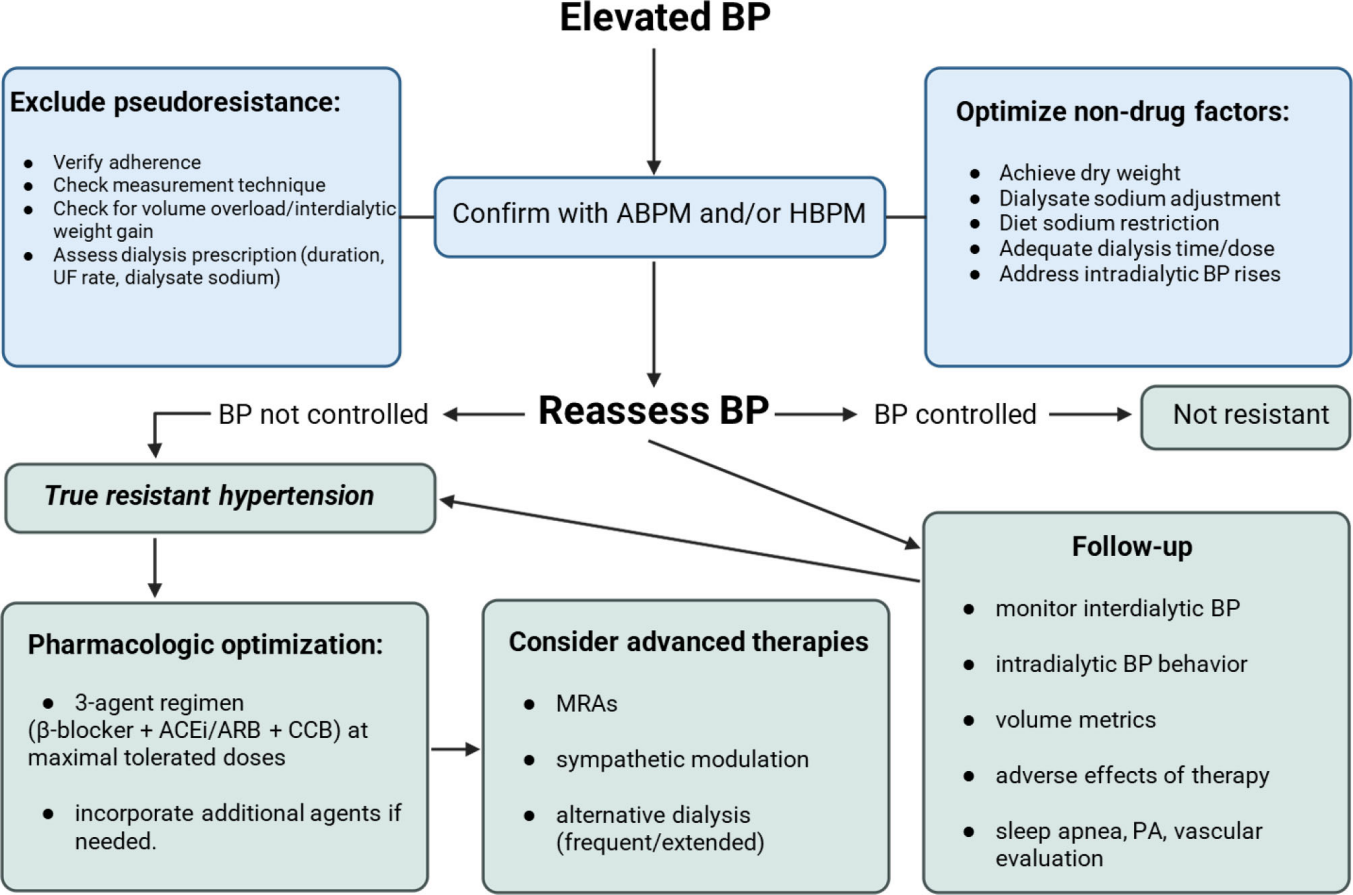
Evaluation and management of resistant hypertension in HD patients.

In summary, an increase in BP during dialysis should prompt a comprehensive assessment of volume management, dialysate composition to minimize sodium load, ultrafiltration profiling to reduce sympathetic activation, as well as consideration of dialysis dose, duration, and antihypertensive timing. These measures are likely to provide more effective management of arHTN than simply adding more drugs (Fig. 4).

## TIP 9: HOW TO MANAGE TRULY RESISTANT HYPERTENSION

Resistant hypertension is highly prevalent among both HD and PD patients, affecting up to 42%, and is often driven by secondary causes such as renovascular disease, chronic glomerulonephritis, polycystic kidney disease, and endocrine disorders like primary aldosteronism [78, 83]. However, the definition of tRH is quite challenging in dialysis patients for several reasons discussed earlier (Tip 8).

The primary therapeutic focus is on achieving optimal volume control through adequate dialysis and dietary sodium restriction, as volume overload is a major modifiable contributor to hypertension in this group [78]. If volume control cannot be achieved using conventional HD methods, extending the duration and/or increasing the frequency of treatment may be a suitable approach, as increasing the frequency of HD has been shown to lower BP and reduce the number of antihypertensive medications required [84]. Overactivation of the SNS is a key contributor and surrogate, leading to increased vascular resistance and sustained hypertension in chronic kidney failure. This sympathetic overactivity is a major target for treating resistant hypertension in chronic kidney failure. While bilateral nephrectomy reduces sympathetic overactivity and thereby BP in individuals with kidney failure or in kidney transplant recipients [85, 86], there are interventions such as catheter-based renal denervation, which aims to reduce BP by disrupting renal sympathetic nerve signalling [87]. Kidney transplantation is the preferred treatment for end-stage renal disease and can improve BP control by restoring renal function and better managing volume status. However, hypertension remains common post-transplant [88].

Aldosterone antagonism, using mineralocorticoid receptor antagonists (MRAs), is increasingly recognized as an important strategy. In resistant hypertension, excess aldosterone promotes inflammation, fibrosis, and sodium retention, exacerbating BP elevation. MRAs can be effective in lowering BP, but their use in dialysis patients especially for reducing CV mortality has not been demonstrated [34, 89]. Newer nonsteroidal MRAs and aldosterone synthase inhibitors are under investigation in nondialysis patients and may offer safer alternatives in the future [90].

In summary, managing resistant hypertension in dialysis patients requires a multifaceted approach: confirming true resistance, optimizing volume status, individualizing pharmacologic therapy, considering advanced interventions like nephrectomy or renal denervation, and addressing neurohormonal contributors such as sympathetic overactivity and aldosterone excess. This comprehensive strategy is essential to improve CV outcomes and quality of life in this high-risk population.

## TIP 10: PRIMUM NON NOCERE!

BP management may be particularly complex in certain subgroups of dialysis patients or specific clinical settings. Older, frail patients and diabetics with autonomic dysfunction often exhibit orthostatic hypotension with a high risk of falls and fractures. Both orthostatic hypotension and falls are associated with increased mortality risk in dialysis patients [91, 92], while recurrent hypotension also predisposes to vascular access thrombosis [93]. These frail individuals may benefit from assessment of standing BP to guide ultrafiltration targets and avoid overuse of antihypertensives [94]. The 2005 KDOQI guidelines suggest a higher supine BP in hypotension-prone diabetic patients to prevent symptomatic postural hypotension [1].

Comorbidities such as heart failure, diastolic dysfunction, or ischemic heart disease can limit cardiac reserve. Additionally, diabetic or age-related autonomic dysfunction may reduce vasoconstrictor responses. Both factors contribute to intradialytic haemodynamic instability [95]. In patients with recent CV events, such as MI, aggressive BP management could lead to intradialytic hypotension (IDH) and precipitate myocardial ischaemia. In a small study of 35 kidney failure patients, there was an increased risk of IDH for patients after acute MI compared to those with angina or stable condition [96].

IDH induces myocardial stunning, characterized by transient regional wall motion abnormalities, progressively leading to adverse cardiac remodelling and heart failure [97]. However, a retrospective cohort study demonstrated that achieving lower postdialysis weight following recent hospitalization correlated with a 40% reduction in pulmonary oedema-related readmissions—the most common cause of rehospitalization—particularly among patients with heart failure [98]. These results underscore the importance of achieving appropriate volume management.

Given the risks associated with IDH, after such an episode careful re-evaluation of dry-weight and volume status should follow, preferably using objective tools, such as bioimpedance spectroscopy or LUS, which can accurately assess volume status and improve haemodynamic stability during dialysis [99, 100]. Routine withholding of antihypertensive medication before dialysis, although a common practise, is currently discouraged due to insufficient evidence [101], with studies demonstrating no difference in the frequency of IDH episodes [35, 102].

## CONCLUSION

The standardization of dialysis care over the past several decades, facilitated by disease management protocols, has led to marked improvements in patient survival, safety, and overall quality of care. However, notable residual mortality risk persists, with annual rates approaching 15% in Europe. Additional risk reduction may be obtained through more individualized treatment approaches, particularly in the area of BP management, which presents a valuable opportunity for personalization within dialysis care. The ten tips outlined above offer a structured framework for achieving this objective.

## Data Availability

No new data were generated or analysed in support of this research.
